# Instrumental Drift in Untargeted Metabolomics: Optimizing Data Quality with Intrastudy QC Samples

**DOI:** 10.3390/metabo13050665

**Published:** 2023-05-16

**Authors:** Andre Märtens, Johannes Holle, Brit Mollenhauer, Andre Wegner, Jennifer Kirwan, Karsten Hiller

**Affiliations:** 1 Department of Bioinformatics and Biochemistry, Braunschweig Integrated Centre of Systems Biology, Technische Universität Braunschweig, 38118 Braunschweig, Germany; 2 Physikalisch-Technische Bundesanstalt, 38116 Braunschweig, Germany; 3 Department of Pediatric Gastroenterology, Nephrology and Metabolic Diseases, Universitätsmedizin Berlin, 13353 Berlin, Germany; 4 Department of Neurology, University Medical Center Göttingen, 37073 Göttingen, Germany; 5 Paracelsus-Elena-Klinik, 34128 Kassel, Germany; 6 Berlin Institute of Health at Charité, Universitätsmedizin Berlin, 10117 Berlin, Germany

**Keywords:** metabolomics, quality control, analytical variation, batch effects

## Abstract

Untargeted metabolomics is an important tool in studying health and disease and is employed in fields such as biomarker discovery and drug development, as well as precision medicine. Although significant technical advances were made in the field of mass-spectrometry driven metabolomics, instrumental drifts, such as fluctuations in retention time and signal intensity, remain a challenge, particularly in large untargeted metabolomics studies. Therefore, it is crucial to consider these variations during data processing to ensure high-quality data. Here, we will provide recommendations for an optimal data processing workflow using intrastudy quality control (QC) samples that identifies errors resulting from instrumental drifts, such as shifts in retention time and metabolite intensities. Furthermore, we provide an in-depth comparison of the performance of three popular batch-effect correction methods of different complexity. By using different evaluation metrics based on QC samples and a machine learning approach based on biological samples, the performance of the batch-effect correction methods were evaluated. Here, the method TIGER demonstrated the overall best performance by reducing the relative standard deviation of the QCs and dispersion-ratio the most, as well as demonstrating the highest area under the receiver operating characteristic with three different probabilistic classifiers (Logistic regression, Random Forest, and Support Vector Machine). In summary, our recommendations will help to generate high-quality data that are suitable for further downstream processing, leading to more accurate and meaningful insights into the underlying biological processes.

## 1. Introduction

The metabolome is a collection of small molecules (<1500 Da) or metabolites that are involved in cellular processes, including energy production, signaling, and synthesis of cellular components. It integrates information from the genome and regulatory processes, as well as environmental factors such as diet and lifestyle. Because the metabolome reflects the downstream effects of these factors on cellular function, it is very close to the actual phenotype of a cell, tissue, or organism. As such, studying the metabolome can provide valuable insights into disease mechanisms, biomarker discovery, drug development, and precision medicine [1,2].

**Figure 1:**
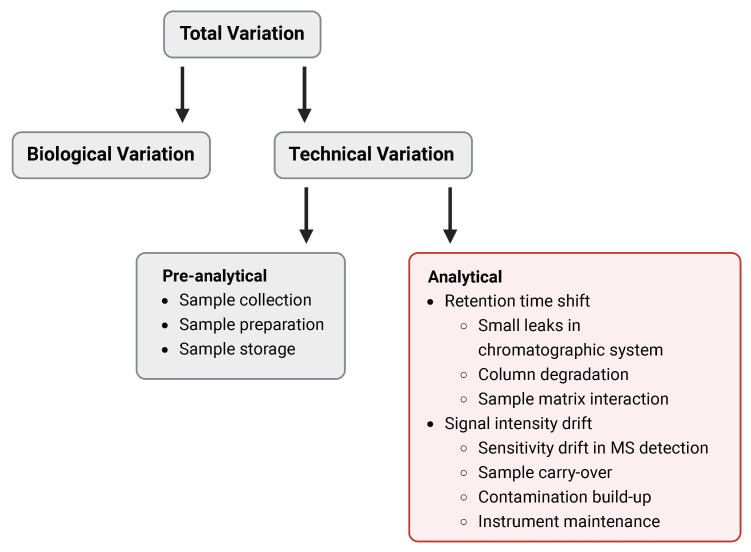
Sources of variation in quantitative metabolomics. The total variation of each data value is comprised of biological, random, and technical variation. Technical variation can be divided in pre-analytical and analytical variation. Pre-analytical variation is induced by either poor methodology or variation in processing during sample collection, preparation or storage. Analytical variation in sample values originates from the analytical technique itself and is reflected in retention time and signal intensity shifts.

The most employed analytical techniques in metabolomics are gas chromatography (GC) and liquid chromatography (LC) coupled to mass spectrometry (MS), both enabling the simultaneous assessment of many metabolites in large cohorts [3,4]. Despite significant technical advances in the field of mass spectrometry, technical variation still remains a challenge, in particular in large clinical cohort studies. Multiple factors introduce technical variation, which is categorizable in pre-analytical and analytical variation (Figure 1).

Pre-analytical variation is introduced by different collection containers, pre-storage preparation, and sample storage conditions. For example, metabolic profiles of blood plasma can be affected by different types of anticoagulant-coated tubes [5,6] as well as different temperatures and time periods of storage due to conversions and degradation of metabolites [7,8,9]. However, even when pre-analytical processing is optimal, technical variation introduced by the analytical platform due to instrumental drifts, can never be excluded (analytical variations). The instrumental drifts during both GC- and LC-MS measurements lead to shifts in retention time (RT) [10,11,12,13,14,15,16] and signal intensity [17,18], both of which need to be considered during data preprocessing. RT is widely used for aligning chromatographic peaks in GC- and LC-MS runs that belong to identical analytes in different samples [19,20,21]. External calibration can be incorporated in the metabolome analysis workflow for analyzing a large number of samples, where RT calibrant runs are carried out every 30 to 40 sample runs to calibrate sample chromatograms between the RT calibrant runs [22,23]. This procedure works most of the time, but there are occasional occurrences, such as small leaks in the chromatography system, minor degradation of column performance, and interactions between different compounds of the analyzed sample matrix, where RT shifts may happen in some samples between the RT calibrants.

The occurrence rate of these unexpected RT shift events increases as the number of analyzed samples increases.

For this task, several alignment algorithms and computer programs are publicly and commercially available, but due to the high complexity of the metabolome, further improvements of existing approaches as well as manual interventions are still needed [22,24,25]. We will introduce one strategy to detect and avoid misalignments due to RT shifts in large studies as part of this review.

Another challenge for large cohorts are significant variations of feature intensities due to instrument drifts; these effects are usually categorized into intra- and inter-batch effects. In this regard, a batch is defined as a set of samples processed and analyzed by the same experimental procedure (same operator and instrument) in an uninterrupted manner. Since the capacity of certain chromatographic equipment (e.g., columns, liners) is limited, cohorts with a higher number of samples are typically partitioned into several batches [26]. Intra- and inter-batch effects occur due to multiple reasons, and a significant source of signal variability is a sensitivity drift over time and across batches in MS detection, as metabolite quantification relies on the intensities of MS peaks. In GC-MS, instrumental causes of changes in intensity between batches mainly occur due to instrument maintenance, ageing, and tuning [27,28]. Of course, machine maintenance, such as the exchange of the liner or column cleaning, is necessary to maintain adequate peak intensities in large cohorts. Another source of technical variation is the problem of sample carry-over and contamination build-up, which could differ between batches [17,29,30]. Sample carry-over is caused by samples containing large amounts of metabolites, from which residuals remain on the column and may affect metabolite signals in later samples of the sequence run. Contamination build-up is caused by compounds trapped in the ion source leading to reduced mass spectral performance. These systemic variations cause detectable differences between samples, which can lead to false discoveries, as batch effects can be stronger than inter-phenotype effects, as highlighted in various studies [18,31,32,33]. Several methods have been developed to tackle this problem, as batch effects in metabolomic experiments are impossible to entirely eliminate. The simplest approach is the randomization of samples within the sequence run. Complete randomization removes the risk of introducing bias, and the variance observed in each biological sample will be a combination of the biological and technical variation. However, when the sample size is so large that the measurement has to be divided into different batches, a blocked approach has to be performed, where only samples within one batch can be compared, reducing the statistical power [34]. Therefore, batch-effects need to be eliminated so that samples between batches can be directly compared. One approach is spiking the samples with labeled internal standards (LIS) as controls. However, in untargeted metabolomics, where all metabolites are of interest, a large number of LIS needs to be added to the samples. This would increase the risk of LIS coeluting with metabolites of interest. Moreover, the added standards may not be representative for the specific chemical characteristics of the unknowns, and response factors may differ. Therefore, spiking with LIS is usually avoided in untargeted metabolomics [26]. The most used and robust methods include the modeling of the above-described batch effects based on intrastudy quality control (QC) samples [21,26,35].

In this review, we outline the importance of incorporating QC samples into the measurement sequence. The review then compares three different methods for adjusting batch effects using QC samples. The first method is a simple and easy-to-implement median-based normalization technique [36], the second method incorporates a regression-based normalization method using a penalized cubic smoothing spline called Quality Control-Robust Spline Correction (QC-RSC) [37]. Finally, a recently published normalization method, Technical variation elimination with ensemble learning architecture (TIGER) [38], is discussed. Our article provides a comparative evaluation of these three strategies using two GC-MS and one LC-MS-based data sets previously recorded in our labs [36,39]. At last, we introduce an effective method for identifying and correcting quantification errors due to peak misalignment. Overall, this review provides a comprehensive guide for researchers to process and analyze untargeted metabolomics data acquired for high sample number cohorts.

## 2. Intrastudy QC-Samples in Metabolomics

The application of intrastudy QC samples has been recognized as a valuable tool to significantly improve the validity of large-scale metabolomics studies [21,26,35,40,41]. The QC samples should reflect the aggregated metabolite composition of all biological samples for a certain study [40,41,42]. Typically, the best way to prepare QC samples is to mix all biological test samples in equal amounts [42], because as such, the QCs are closest to the biological samples in means of composition (same sample matrix and metabolites). If the amount of material is limited or sample preparation starts before the last sample has been collected, it is not possible to generate sufficient QCs. To mitigate this, commercially available QC samples can mimic the composition of the biological samples, although to a lower accuracy compared to intrastudy QC samples [43,44,45]. Dunn et al. used commercially available serum samples, but had to remove 20% of all features, due to differences in metabolic composition between the commercial QC sample and samples from the study population. Therefore, preparing intrastudy QC samples from a representative subset of biological samples is the better solution [44]. Another option is generating intrastudy QCs from the same sample type but from another biological source. A clear disadvantage in this case is that the metabolite concentrations and the sample matrix differ from the biological samples. As a last option, artificial QCs can be created with chemical standards, whereas as many metabolites from as many metabolite classes as possible are dissolved in a dummy matrix [46].

There are three major reasons to employ QC samples: The first is the initial equilibration of the measurement system. Each sequence of samples should start with conditioning QC samples. In the case of GC-MS and LC-MS, usually the data of the first four to eight injections are not stable [47,48,49]. This effect occurs especially in the context of preventative maintenance, after which active sites of the column are not equilibrated or blocked with the sample matrix. Multiple injections of the QCs prior to the main sample acquisition will condition the column sufficiently [44,47,50]. The actual number of required conditioning samples depends on several factors, particularly sample type, chromatographical system, injection volume, chromatographic column, and mass spectrometric design. It has been suggested that each laboratory should determine an individual optimal number of conditioning samples by injecting up to 50 intrastudy QC samples until reproducible results will be acquired [46]. It is to note that the only intention of the conditioning samples is for column conditioning and not for later batch-effect correction.

The second reason to employ QC samples is the evaluation of measurement precision. As all QCs are equal in terms of metabolite concentration and sample matrix, quantitative quality criteria such as the relative standard deviation (RSD) and Dispersion-ratio (D-ratio) can be determined for quality assessment.

The third and most important reason is the modeling and correction of systemic error. Since QC samples are measured intermittently throughout the whole sequence run, changes in instrument performance can be accurately monitored. Data of QCs quantitatively reveal gradual changes in instrument sensitivity, which is extremely useful for the elimination of batch variations. For this, it is crucial to include a sufficient number of QCs into the sequence to maximize the performance of batch-effect correction and lower the risk of overfitting [51]. On the other hand, injecting too many QCs will significantly extend analysis time, which results in an even more pronounced instrumental drift [26,52]. Kamleh et al. quantitatively evaluated the effect of QC frequency on the reproducibility of metabolic features. The number of reproducible features (RSD < 30% and <15%) was only 1.5% and 5% lower when comparing data corrected with a QC injection every 10th sample with data corrected with a QC injection every fifth sample [53]. Therefore, one QC sample should be injected for every third to 10th biological sample [44,53]. Additionally, it is recommended to append two QCs each at the beginning and end of the sequence to avoid extrapolation during batch-effect correction in case of injection failure [40]. If both QCs run successfully, only one of these QCs is used for batch-effect correction, e.g., the first and the last one.

## 3. Methods to Correct Metabolomics Data for Batch Effects

Besides just detecting gradual changes in instrument sensitivity, QC samples are also used for the correction of these effects. In general, such a correction is performed as follows: As batch effects are metabolite specific, each recorded metabolite level needs to be analyzed and corrected, separately. The QC recordings for each metabolite define a pattern of instrument-related signal changes as a function of the injection order. Because all biological samples are flanked by corresponding QCs, it can be assumed that instrument-related signal alterations in the QCs also apply to the neighboring biological samples. This is the reason why a mathematical model can be employed to predict batch variation based on the information of the QCs. The predicted batch variability is then subtracted from the original data to yield batch-effect-free data. A qualified QC-based correction does not only account for inter- and intra-batch effects but is also resistant to overfitting. This is important in order to deter the model from accounting for random variations in the data, leading to overoptimistic quality measures, but non-usable data. In the following, we review and compare three often applied correction methods in regard to their effectiveness for batch-effect removal.

### 3.1. Median Normalization

**Figure 2:**
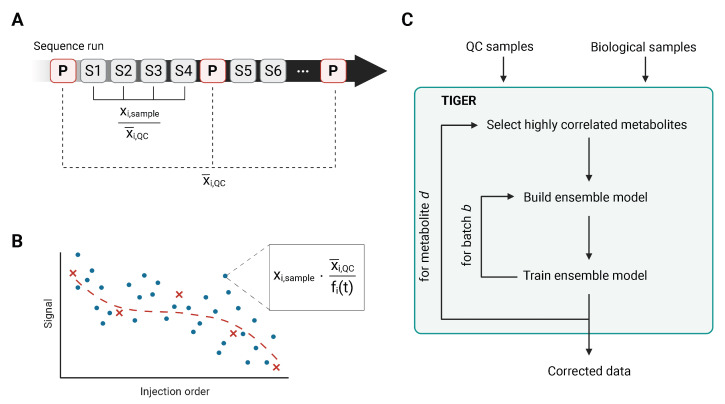
(**A**) Graphical representation of the median-based normalization. For each sample, the median of the three nearest pools is calculated for each metabolite *i*. Then, the metabolite intensity for metabolite *i* is divided by the corresponding median ${\bar{x}}_{i,QC}$ of the pools. (**B**) Schematic functionality of the QC-RSC algorithm. For a given metabolite peak, batch effects can be visualized by plotting the metabolite signal against the injection order. Here, the blue circles represent the biological samples and the red crosses represent the QC samples with which the unweighted cubic smoothing spline is fitted (red dashed line). Then, each sample is normalized by multiplication with the correction factor. The correction factor is the quotient of the median signal of the QCs and the value given by the cubic smoothing spline at injection order *t* of the sample to be corrected. (**C**) Schematic representation of the TIGER algorithm. The TIGER algorithm can be described in three steps. 1. Variable selection, 2. model construction, and 3. data correction.

The first batch-effect correction method is the simplest to apply and just normalizes each sample metabolite signal ($x_{i,sample}$) by the corresponding median signals of neighbouring QCs ($x_{i,QC}$). Specifically, the three in terms of acquisition time and chronologically closest QC samples for each biological sample are chosen. The median signal for each metabolite *i* is calculated based on these three QC samples (${\bar{x}}_{i,QC}$) and is then applied to normalize the metabolite signal of the sample ($x_{i,sample}$). Using the median instead of the mean makes this approach more robust against outliers or missing (zero) values within the QCs (Figure 2A).

### 3.2. Quality Control-Robust Spline Correction

Quality Control-Robust Spline Correction (QC-RSC) is an advanced regression-based method [37]. For this method, an unweighted cubic spline *f* is fitted to the QC data ($x_{QC}$) as a function of the injection order ($t_{QC}$), with *n* being the length of $t_{QC}$ (Equation (1)). In contrast to non-parametric models, QC-RSC has the advantage of accounting for more complex batch-related variations in metabolite signals. Furthermore, compared to the Quality Control-Robust LOESS Signal Correction algorithm [44], QC-RSC is computationally more efficient, by replacing the two-step LOESS QC fitting and piece-wise polynomial regression stage with a single-step adaptive cubic smoothing spline algorithm.

The spline *f* minimizes the distance between model fit and QCs under consideration of a roughness penalty, controlled by the smoothing parameter *p* (0<p<1). The roughness penalty penalizes the variability in the function *f*, with p→0 resulting in an interpolating spline and p→1 in a linear least squares regression.

(1) $$p\sum_{i=1}^{n}{\left(x_{QC}\left(i\right)-f\left(t_{QC}\left(i\right)\right)\right)}^{2}+\left(1-p\right)\int {\left(\frac{d^{2}f}{dt_{QC}^{2}}\right)}^{2}dx$$

To avoid overfitting, *p* is optimized using leave-one-out cross-validation. Each metabolite is then normalized by its own correction function, which removes intra-batch effects. To remove inter-batch effects, the value is furthermore divided by the median signal of the metabolite of the intrastudy QC samples (Figure 2B).

### 3.3. Technical Variation Elimination with Ensemble Learning Architecture

TIGER (Technical variation elImination with ensemble learninG architEctuRe) is the most sophisticated algorithm for the batch-effect correction discussed here. It is an adaptable ensemble learning architecture comprised of several base models [38]. The TIGER algorithm starts by selecting metabolites highly correlated with the objective metabolite, which will be the features for the ensemble model. The ensemble model is constructed for each batch separately and consists by default of *n* Random Forest (RF) models with different hyperparameter combinations, chosen from a hyperparameter pool defined by the user. The Random Forest model is trained on data comprised of error ratios $y^{\prime }$ of the objective metabolite and the raw metabolite signals of the correlated metabolites *X*. The error ratio is calculated as follows:

(2) $$y^{\prime }=\frac{y-\bar{y}}{\bar{y}}$$

Here, *y* denotes the raw signal of the objective metabolite and $\bar{y}$ the median of *y* across the whole data set. The model’s performance is evaluated in a *K*-fold cross validation with a loss function of $L(y,y^{\prime })$,

(3) $$L\left(\hat{y},y^{\prime }\right)=\frac{1}{K}\sum_{k=1}^{K}\frac{\left|{\hat{y}}^{\left(k\right)}-y^{\prime (k)}\right|}{y^{\prime (k)}}$$

where ${\hat{y}}^{\left(k\right)}$ is the predicted error ratio and $y^{\prime (k)}$ the actual error ratio of the *k*th CV fold. Based on the loss function, the base model will receive a weight, such that high-performing models have high weights and under-performing models have low weights, but its information is still considered.

(4) $$w_{i}=\frac{exp(-L\left({\hat{y}}_{i},y^{\prime }\right))}{\sum_{n}^{i}exp(L\left({\hat{y}}_{i},y^{\prime }\right)}$$

Here, *n* is the number of all base models, and *i* is the *i*th base model. For the actual data correction, the base models are retrained on the whole data set. The error ratios $y^{\prime }$ and metabolite signals *X* are used to train the RF model. Hence, the RF model will also predict error ratios, which need to be converted back to metabolite signals. The final result of the algorithm is the weighted sums of all base models (Figure 2C).

By selecting only a small set of features and the RF algorithm, TIGER’s base models are of moderate complexity, thus mitigating the risk of capturing random noise in addition to the technical variation. Moreover, an ensemble learning architecture is employed, further improving the models’ robustness and lowering the risk of overfitting by considering the output from strong as well as weak models. Altogether, the TIGER algorithm prioritizes robustness and high generalization over high complexity, making this a valuable method even for data with small sample sizes or data with a high degree of noise.

## 4. Evaluation of Batch-Effect Correction Methods

### 4.1. Evaluation Metrics

To evaluate the performance of the different batch-effect correction methods, we employed three different quantitative quality criteria, namely the RSD and D-ratio for each metabolite, as well as the Euclidean distance of QCs after the principal component analysis (PCA). The RSD is a widely used metric, which is calculated for each metabolite *i* within the QCs by dividing the standard deviation $\sigma_{i,QC}$ by the arithmetic mean ${\bar{m}}_{i,QC}$. This leads to a unitless and standardized measure comparable among all detectable metabolites.

(5) $$RSD_{i,QC}=\frac{\sigma_{i,QC}}{{\bar{m}}_{i,QC}}\cdot 100\%$$

A typically accepted RSD threshold for metabolites in biomarker discovery should be below 20% for LC-MS and below 30% for GC-MS [18,33,44,47,48]. However, only observing the RSD may result in over-optimistic results, as batch-effect correction methods remove the batch effects based on the QCs. This way, an over correction of the data, which could lead to the removal of the biological variation, would not be detected. Thus, we additionally applied the relation of the statistical dispersion of the QCs to the dispersion of the biological test samples to evaluate the normalization performance [54,55]. The D-ratio is calculated by dividing the technical variation by the total observed variation; this is the sum of technical and biological variation. Here, the variance of the QCs ($\sigma_{i,QC}^{2}$) approximates the technical variation, and the variance of the biological test samples ($\sigma_{i,sample}^{2}$) approximates the overall biological variation [46].

(6) $$D\text{-}ratio_{i}\approx \frac{\sqrt{\sigma_{i,QC}^{2}}}{\sqrt{\sigma_{i,sample}^{2}+\sigma_{i,QC}^{2}}}\cdot 100\%$$

A D-ratio close to 0% would be a perfect measurement, where the technical variance is zero and all observed variance originates from the biological variation. On the other hand, a D-ratio of 100% would be the worst possible measurement, where there is no biological variation and only noise is detected. A metabolite, where $\sigma_{i,sample}^{2}\gg \sigma_{i,QC}^{2}$ with a D-ratio below 50%, is preferred [55].

Another method to visually evaluate batch-effect-removal is the PCA. By plotting the first two principal components, the clustering of batches and the removal of those can be observed. High quality data show tightly clustered QC data points at the origin of the PCA and equally distributed sample points around the QCs. The Euclidean distance between the centroid of the QCs, and each QC sample point can be calculated to quantitatively assess the effect of the batch-effect correction on the QCs itself.

(7) $$d\left(C_{QC},x_{i,QC}\right)=\sqrt{{\left(C_{QC,PC1}-x_{QC,PC1}\right)}^{2}+{\left(C_{QC,PC2}-x_{QC,PC2}\right)}^{2}}$$

Here, $d(C_{QC},x_{i,QC})$, represents the Euclidean distance between the centroid of all QC samples $C_{QC}$ and each individual QC sample $x_{i,QC}$ in the PCA. Each sample point as well as the centroid have two coordinates, which are denoted as the principal components PC1 and PC2, respectively. The Euclidean distance of QCs is frequently used to evaluate the analytical variability [30]. These three performance measures provide an in-depth picture for evaluating the batch-effect correction methods.

### 4.2. Comparison of Batch-Effect Correction Methods

To provide recommendations on which method to choose, we evaluated all of the three above highlighted batch-effect correction methods and performed RSD, D-ratio, and a PCA. Furthermore, the methods were applied to normalize GC-MS and LC-MS data sets to evaluate differences in measurement techniques.

**Figure 3:**
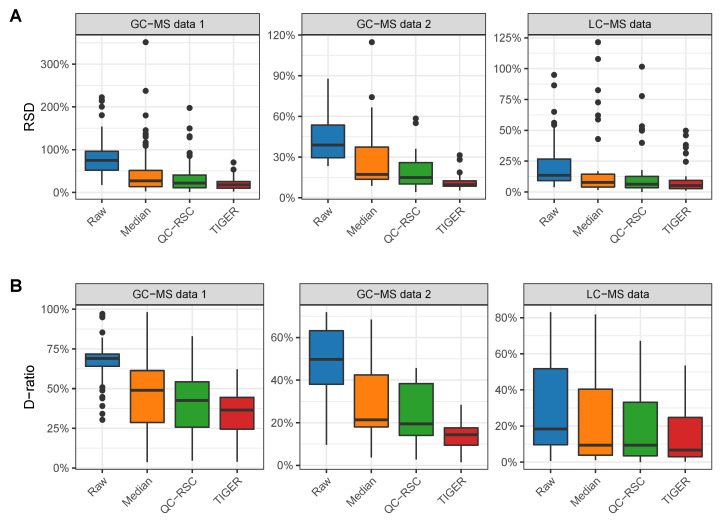
Comparison of normalization methods. A total of 140 metabolic features are present in GC-MS data 1, 25 in GC-MS data 2, and 42 in the LC-MS data. (**A**) Distribution of RSD of QC samples before and after normalization. (**B**) Distribution of D-ratio before and after normalization. Outliers are shown as black dots.

All batch-effect correction methods reduced the technical error in each tested data set, as demonstrated by the reduced RSD as compared to the raw data (Figure 3A) . Here, TIGER performed best for each data set with a reduction of the median RSD by more than 60%. QC-RSC reduced the median RSD by more than 50% and the median normalization by more than 40% for each data set. Furthermore, all methods were able to reduce the D-ratio (Figure 3B) . Again, TIGER performed best by reducing the median D-ratio by 71%, 64%, and 43% for GC-MS 1 and 2 data and the LC-MS data, respectively. For the GC-MS 1 data, QC-RSC performed better than the median normalization, with a reduction of the D-ratio by 41% compared to 27%. For GC-MS data 2 and the LC-MS data, QC-RSC and the median normalization performed equally good by reducing the D-ratio by approximately 50%.

**Figure 4:**
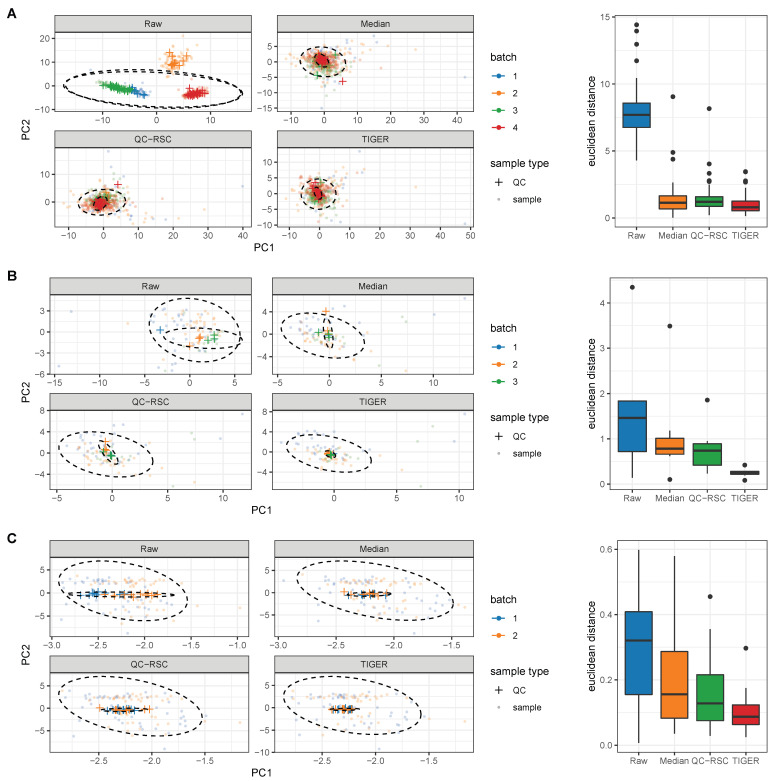
PCA plots of different normalization methods for (**A**) GC-MS data 1, (**B**) GC-MS data 2, and (**C**) LC-MS data. QCs are represented as crosses and biological samples as dots, which are partially transparent. Box plots show the euclidean distance of the QCs to its centroid within the PCA plots. Outliers are shown as black dots.

To further evaluate the different methods, we performed a PCA. As described in the previous chapter, we expected all QC samples to cluster tightly together after removing the batch effects, while we expected all biological samples to be distributed across the plot. Figure 4 depicts the PCA plots for the raw data and the batch-effect correction methods.

The PCA of the raw data clearly partitioned the QCs into the underlying batches for every data set, with GC-MS 1 having the most substantial batch effects. After batch-effect correction, all methods resulted in similar plots with tightly clustered QC samples at the origin of the PCA plot scores and equally distributed biological samples. Therefore, all methods were able to eliminate batch effects regardless of the analytical technique and led to a small analytical variation relative to the biological variation. To quantitatively analyze the PCA plots, we calculated the Euclidean distance between each QC sample point and its corresponding centroid (Figure 4). The Euclidean distance was reduced with all batch-effect correction methods for each data set compared to the raw data. Here, QC-RSC and the median normalization perform similarly for all data sets with a median euclidean distance of approximately 1.2 for GC-MS 1, 0.75 for GC-MS 2, and 0.13 for LC-MS data. The TIGER normalization achieves the lowest distance, which is slightly better than QC-RSC and median normalization. All methods were able to reduce the technical error without overfitting and keeping the biological variation intact by reducing both RSD and D-ratio of the QC samples. Additionally, the removal of batch effects could be observed in the PCA plots and by calculating the Euclidean distances of the QCs. For all quality criteria, TIGER outperformed all other methods.

However, it is important to note, that the previously described evaluation criteria are all based on the same QCs used to fit and train the batch-effect correction methods. This could lead to overoptimistic results; furthermore, it does not measure the impact of the batch-effect correction on the biological samples. For this purpose, different machine learning classifiers were trained to classify persons into Parkinson’s disease or healthy control groups based on a published cerebrospinal fluid metabolomics data set to test the power of these algorithms in terms of biological information [36]. Before training the machine learning classifiers, the three batch-effect correction methods had been applied to normalize the data based on intermittently recorded QCs. The stratification performance of these classifiers indicates whether a batch-effect correction method reduces or increases the predictability of phenotypes by interfering biological information. After correction for batch effects by the three methods, we at first removed metabolites that were not accepted by our quality criteria (RSD < 30% and D-ratio < 50%). For the median normalization, 52 metabolites passed the quality acceptance criteria (37.1% of all detected metabolites), 66 for QC-RSC (47.1% of all detected metabolites), and 103 for TIGER (73.6% of all detected metabolites).

**Figure 5:**
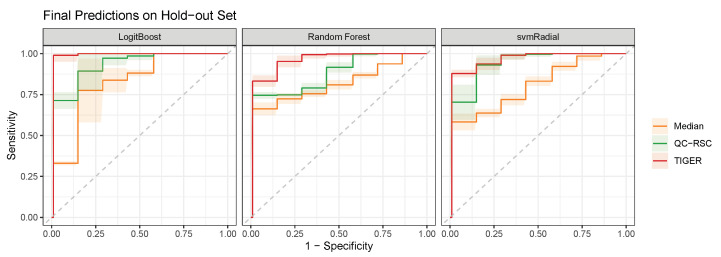
Results of machine learning classifier on corrected data sets. The performance of three probabilistic classifiers were evaluated on predicting the phenotype based on the metabolic signature of CSF samples. The classes of Parkinson’s disease and the healthy control had to be predicted. For all three classifiers, the models trained on the TIGER-corrected data achieved the highest AUROC (0.979±0.021 for LogitBoost, 0.969±0.009 for RF, and 0.958±0.016 for svmRadial), followed by the models trained on data corrected by QC-RSC (0.963±0.019 for LogitBoost, 0.893±0.025 for RF, and 0.889±0.026 for svmRadial). The models trained with the median-corrected data demonstrated the worst performance (0.760±0.008 for LogitBoost, 0.841±0.011 for RF, and 0.820±0.021 for svmRadial).

Next, we selected the metabolic features to train the model on using recursive feature elimination (RFE). This method trains the model on the full number of features and assigns each feature an importance metric. The least important features are removed, and the process is repeated until a pre-defined number of features will be reached. Here, ten features were selected for each corrected data set by RFE, resulting in three sets of features. Citramalate, pyroglutamate, tryptophan, urea, and glycine were present in all three feature sets and, finally, were used to train the classification models. Here, we focus on three popular probabilistic classifiers, namely boosted Logistic Regression (LogitBoost), Random Forest (RF), and radial kernel Support Vector Machine (svmRadial). For the optimal evaluation of the classifiers, we performed a double repeated cross-validation (CV) approach with two loops. The outer loop generates 100 random splits of training and hold-out sets. The inner loop is used to tune the models’ hyperparameters on the training data by maximizing the Area under the Receiver Operating Characteristic curve (AUROC) with repeated CV (10 repeats, 5 folds). The model with the highest AUROC in the CV then predicts the patients’ class of the hold-out set. The results of the predictions is shown in Figure 5. Based on the ROC curve evaluation, the models trained on the TIGER corrected data reached the highest AUROC for each classifier (0.979±0.021 for LogitBoost, 0.969±0.009 for RF, 0.958±0.016 for svmRadial), followed by the models trained on the data corrected with QC-RSC (0.963±0.019 for LogitBoost, 0.893±0.025 for RF, and 0.889±0.026 for svmRadial). The worst performance was observed for the classifiers trained on the median-corrected data (0.760±0.008 for LogitBoost, 0.841±0.011 for RF, and 0.820±0.021 for svmRadial).

In summary, TIGER performed best for the batch-effect correction in our evaluation reflected in the lowest RSD, D-ratio, and median Euclidean distance of QC samples in the PCA. This was also the reason why many more metabolites passed our predefined quality criteria and remained in the data set for further analysis. In addition, the performance of machine learning classifiers trained on the data set corrected with TIGER demonstrated the best performance, emphasizing that this batch-effect correction method captures the inter-phenotype information present in the data in the most optimal way. All in all, we recommend using TIGER as the preferred method for the batch effect correction due to the minor loss of metabolic information. On the other hand, QC-RSC is in the advantage for the normalization of very large cohorts, as the computation costs of TIGER are relatively high.

## 5. Advanced Strategies to Further Improve Metabolite Quantification and Chromatogram Alignment

A prerequisite for the successful application of the above discussed batch-effect correction algorithms is an accurate chromatogram alignment and picking of quantification peaks. The correct alignment of metabolite features across measurements is crucial for an accurate quantification in the context of every metabolomics’ analysis. RT shifts of molecular features within- and between batches can result in wrong alignments of metabolite features, especially for closely eluting metabolites with similar or even identical mass spectra, such as isomers. In the following, we present a strategy to screen and to correct for quantification errors due to metabolite feature misalignments. We take advantage of the fact that EI ionization generates highly reproducible fragmentation patterns for a certain metabolite. We assume that an increased or decreased metabolite amount in the sample affects all fragment ions equally, and that the same applies for a potential drop in instrument sensitivity over time. Hence, the ratio of a pair of fragment ion intensities must be identical for a certain metabolite over all measurements and independent of the sample or instrument condition. As a demo data set, we chose a previously published GC-MS-based metabolomics data set that was recorded for CSF samples in the context of Parkinson’s disease [36].

**Figure 6:**
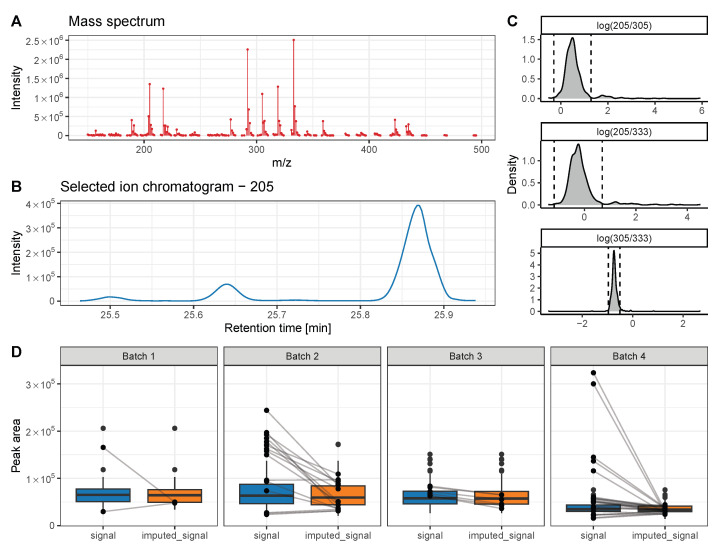
Identification of quantification errors in chromatograms of gluconic acid. (**A**) Mass spectrum. (**B**) Selected ion chromatogram of mass 205. (**C**) Distribution of quantification ion ratios. Vertical dotted lines depict the threshold of accepted values, which is calculated by the median signal intensity ± three times the MAD. (**D**) Box plots of raw metabolite signals and imputed signals, based on outlier detection.

Within this data set, gluconic acid elutes at 25.65 min and the peak integrals of fragment ions 205, 305, and 333 were automatically assigned for quantification (Figure 6A). However, two coeluting compounds produce ion chromatographic signals on SIC 205 at 25.5 and 25.88 as well (Figure 6B). Due to the close elution of these metabolites, there is a substantial risk of picking the wrong peaks of this SIC for integration during automatic data processing. Integrating the wrong peak of this SIC results in either lower or higher log-fold changes of ion ratios, which can be indicative for a misalignment if laying outside the determined outlier threshold (Figure 6C). In such a case, outlier values can either be removed from the data set or imputed based on the quantification of the other metabolite ions (here 305 and 333). Depending on the type of missing values, different imputation methods should be chosen for optimal results. Here, the values are missing at random (MAR), due to suboptimal data preprocessing. Therefore, we chose the Random Forest imputation method that performed best for MAR [56,57]. For a detailed evaluation of imputation methods with further types of missing values, we refer the reader to the paper of Wei et al. [57]. By taking advantage of this approach, we identified 48 falsely picked peaks for gluconic acid out of 600 total measurements (Figure 6D). The imputation of metabolite signals based on the other metabolite ions decreased the RSD for this metabolite by 12%.

## 6. Conclusions and Future Directions

In this article, we reviewed and evaluated three popular algorithms suited for the elimination of mass spectrometric noise based on intermittently measured QC samples in metabolomics studies with a high number of samples. Furthermore, we introduced a strategy to improve chromatogram alignment and peak picking in GC-MS data. To increase the quality of large metabolomics studies, an optimal workflow should include the following steps: 1. Preparation of adequate QCs is essential for bigger cohorts. 2. The sequence run should start with five to ten QC samples solely for equilibration of the analytical system. 3. The sequence of sample measurements should be random and at least one QC sample should be measured in-between three to seven samples. In addition there should be two QC samples at the sequence beginning and end. 4. For high sample numbers, the sample blocks between QCs should be increased to avoid an unnecessary extension in analysis time. For lower sample numbers, the sequence should contain at least eight QCs (without the conditioning QCs). After data acquisition, the peak picking, integration and mapping of metabolites should be verified. For this, we propose an easy-to-implement method to check the quantification of metabolites based on log-fold changes of the quantification ions’ intensities. To advise which method to employ for the batch effect removal, we evaluated three different methods: a median-based approach, QC-RSC, and TIGER. Although all three methods significantly removed batch effects and drifts in instrument sensitivity, TIGER always outperformed the other two methods. For this reason and despite the high computational cost of this algorithm, we advise using TIGER. Many more metabolites passed the quality criteria (RSD < 30% and D-ratio < 50%), and overall separation between tested phenotypes was more evident, as highlighted in the better performance of employed machine learning classifiers.

Untargeted metabolomics is an important tool for biomarker discovery, drug development, and precision medicine. These fields rely heavily on large data sets to provide the needed statistical power. Here, the machine learning approach with an ensemble learning architecture has been proven to be the most promising tool for batch-effect correction. Therefore, the performance of batch-effect correction could be further improved by applying deep learning approaches. Furthermore, this could enable the comparison of untargeted metabolomic studies between instruments, which opens the possibility of mining large databases across studies. Potentially, this could lead to the identification of biological mechanisms or biomarkers that would otherwise be hidden in smaller individual studies.

## Data Availability

No new data were created or analyzed in this study. Data sharing is not applicable to this article.

## Abbreviations

The following abbreviations are used in this manuscript:

AUROC	Area under the Receiver Operating Characteristic
CSF	Cerebrospinal fluid
CV	Cross validation
D-ratio	Dispersion-ratio
GC	Gas chromatography
LC	Liquid chromatography
LogitBoost	Boosted Logistic Regression
MS	Mass spectrometry
PCA	Principal component analysis
QC	Quality control
QC-RSC	Quality Control-Robust Spline Correction
RF	Random Forest
RFE	Recursive feature elimination
ROC	Receiver Operating Characteristic
RSD	Relative standard deviation
RT	Retention time
svmRadial	Radial Kernel Support Vector Machine
TIGER	Technical variation elImination with ensemble learninG architEctuRe
